# Supplementary material for: In Vitro High-Throughput Genotoxicity Testing Using γH2AX Biomarker, Microscopy and Reproducible Automatic Image Analysis in ImageJ—A Pilot Study with Valinomycin
Source: Toxins (Basel). 2023 Apr 1;15(4):263. doi: 10.3390/toxins15040263 (PMC10146355; doi:10.3390/toxins15040263)
Supplement: Supplementary file 1 [file toxins-15-00263-s001.zip › toxins-2287007-supplementary.pdf]

Supplementary Materials

Table S1: p-values after 4h treatment.

Cell line	Sample	Well 1	Well 2	Well 3
CHO-K1	Ctrl	0	0	0
	Val30	3.65E-132	3.65E-132	7.43E-205
	Val15	3.90E-107	2.97E-177	2.20E-155
HeLa	Ctrl	0	0	0
	Val30	0	0	0
	Val15	0	0	0

Table S2: p-values after 24h treatment.

Cell line	Sample	Well 1	Well 2	Well 3
CHO-K1	Ctrl	4.84E-220	7.61E-309	5.07E-200
	Val30	7.44E-201	5.48E-186	2.57E-179
	Val15	2.74E-229	2.51E-301	2.91E-206
HeLa	Ctrl	0	0	0
	Val30	0	0	0
	Val15	0	0	0

Null hypothesis: Data comes from a normal distribution
If p-value < 0.001 then null hypothesis can be rejected.

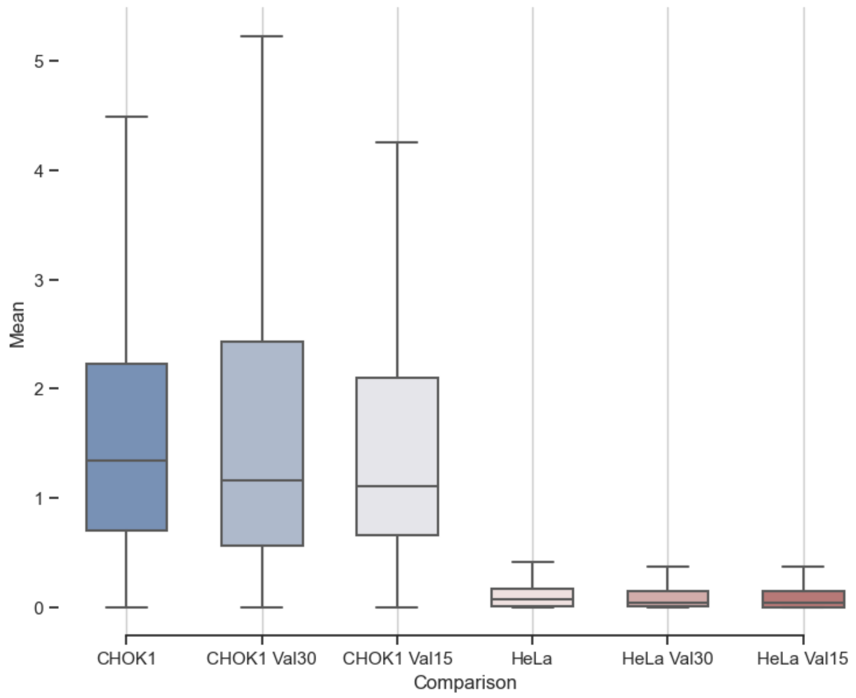

Figure S1. Mean brightness of detected ROI after 4 h. Error bars stand for interquartile range.

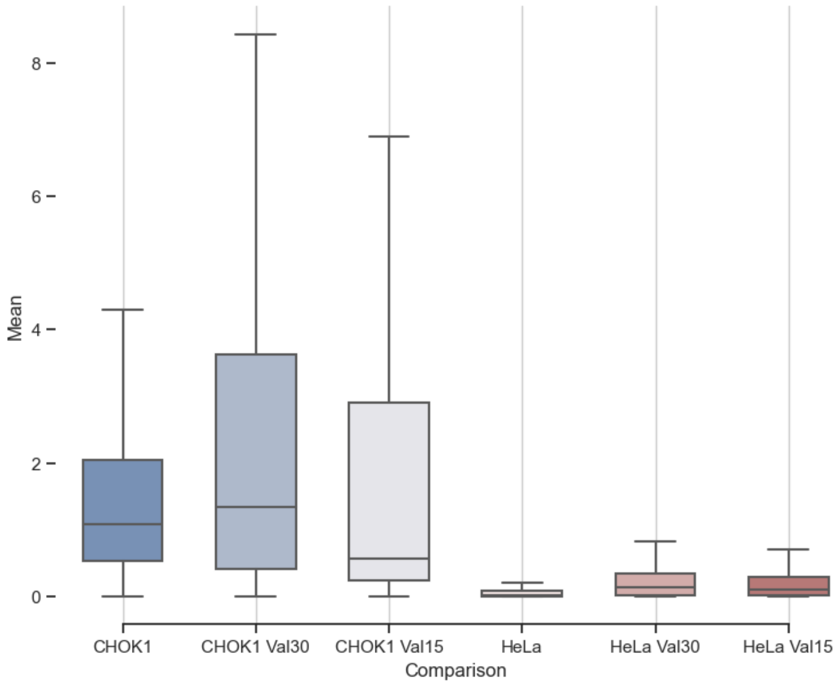

Figure S2. Mean brightness of detected ROI after 24 h. Error bars stand for interquartile range.
